# Opportunities and challenges of active immunotherapy in dogs with B-cell lymphoma: a 5-year experience in two veterinary oncology centers

**DOI:** 10.1186/s40425-019-0624-y

**Published:** 2019-06-07

**Authors:** Laura Marconato, Luca Aresu, Damiano Stefanello, Stefano Comazzi, Valeria Martini, Roberta Ferrari, Fulvio Riondato, Nicole Rouquet, Patrick Frayssinet, Silvia Sabattini

**Affiliations:** 1 Centro Oncologico Veterinario, Sasso Marconi, via San Lorenzo ¼, 40037 Sasso Marconi, Bologna, Italy; 2 Department of Veterinary Science, University of Turin, Grugliasco, Turin, Italy; 3 Department of Veterinary Medicine, University of Milan, Milan, Italy; 4 Urodelia, St Lys, Toulouse, France; 5 Department of Veterinary Medical Sciences, University of Bologna, Bologna, Italy

**Keywords:** Dog, Lymphoma, Spontaneous cancer, DLBCL, MZL, Follicular lymphoma, Cancer immunotherapy, Autologous vaccine, Prognosis

## Abstract

**Background:**

Pet dogs spontaneously develop lymphoma. An anthracycline-based multidrug chemotherapy regimen represents the treatment cornerstone; however, cure is rarely achieved. We have been treating dogs with B-cell lymphoma with an autologous vaccine (APAVAC®) and CHOP-based chemotherapy since 2011.

**Methods:**

To better characterize the safety and efficacy of APAVAC®, and to find the best candidates for immunotherapy, we designed a retrospective study on all dogs treated with chemo-immunotherapy to date and compared them with those dogs treated with chemotherapy only. All dogs were completely staged and re-staged at the end of treatment. The primary endpoint was the effectiveness of chemo-immunotherapy, measured as time to progression (TTP), lymphoma-specific survival (LSS), and 1-, 2-, and 3-year survival rates. The secondary objective was safety.

**Results:**

Three hundred dogs were included: 148 (49.3%) received chemotherapy and 152 (50.7%) chemo-immunotherapy. Overall, the latter survived significantly longer (median LSS, 401 vs 220; *P* <  0.001).

Among dogs with diffuse large B-cell lymphoma, the 1-, 2- and 3-year survival rates were 20, 13 and 8% for chemotherapy, and 51, 19 and 10% for chemo-immunotherapy. The benefit of chemo-immunotherapy was particularly relevant in dogs with concurrent high serum LDH, stage V, substage a disease and not previously treated with steroids (median LSS, 480 vs 85 days; *P* <  0.001). Among dogs with nodal marginal zone lymphoma, those having at least 3 of the aforementioned characteristics significantly benefited from chemo-immunotherapy (median LSS, 680 vs 160 days, *P* <  0.001). The 1-, 2- and 3-year survival rates were 30, 16 and 10% for chemotherapy, and 55, 28 and 10% for chemo-immunotherapy. Among dogs with follicular lymphoma, lack of immunotherapy administration was the only variable significantly associated with increased risk of tumor-related death. Chemo-immunotherapy was remarkably well tolerated, with no local or systemic adverse events.

**Conclusions:**

Overall, the addition of immunotherapy to a traditional CHOP protocol is associated with improved outcome in dogs with B-cell lymphoma, regardless of histotype and evaluated prognostic factors. Moreover, the identikit of the best candidate for immune-therapy was delineated for the most common histotypes. The study also confirms the excellent tolerability of the vaccine.

**Electronic supplementary material:**

The online version of this article (10.1186/s40425-019-0624-y) contains supplementary material, which is available to authorized users.

## Background

Pet dogs most often spontaneously develop B-cell lymphoma [1], for which survival without treatment is measured in weeks or months. Anthracycline-based multidrug chemotherapy represents the cornerstone for the treatment of canine B-cell lymphoma across all disease stages; however, the majority of dogs eventually relapses and develops chemo-resistance, and cure is rarely achieved [2].

Immunotherapy represents a powerful way to treat cancer, and the last decade has witnessed unprecedented advancements in the understanding of both the molecular drivers of lymphomagenesis and mechanisms by which lymphoma circumvents anti-tumor immunity [3, 4].

In oncology, therapeutic vaccines have the theoretical ability to generate cytotoxic T-lymphocytes that reject cancer cells, and memory cells that prevent relapse [5, 6].

Active immunotherapy consisting of hydroxylapatite ceramic powder and HSPs purified from the dogs’ tumors (APAVAC®) has shown promising results in a pivotal placebo-controlled, randomized clinical trial in dogs with DLBCL [7]. Within the first few years of its introduction to the European market, the investigational use of APAVAC® was extended to indolent B-cell lymphomas with suggestive signals of potential usefulness [8].

The aim of this retrospective study was to compare the efficacy and safety of chemotherapy alone versus chemo-immunotherapy in dogs with multicentric B-cell lymphoma of various histotypes in 5 years of clinical experience. The survival advantage of chemo-immunotherapy was further assessed after stratifying dogs according to potential prognostic variables, in order to identify the best candidates for vaccine administration.

Pooling the available clinical data should provide an accurate evaluation of the efficacy of chemo-immunotherapy in canine B-cell lymphoma compared with traditional chemotherapy, posing the basis for future clinical trial design and informing human trials.

## Material and methods

Medical records were reviewed for every consecutive canine patient with previously untreated multicentric B-cell lymphoma that presented to the Centro Oncologico Veterinario (Sasso Marconi, Italy) and Department of Veterinary Medicine (University of Milan, Italy) from 2013 to 2018. This study was prospectively conceived and clinical data on the efficacy and toxicity of treatments were analyzed retrospectively, thus the inclusion criteria were established a priori and all dogs were treated contemporaneously.

To be eligible for inclusion, dogs were required to undergo a complete initial staging work-up and, whenever possible, surgical removal of a peripheral LN to obtain a histopathological diagnosis and material for the vaccine generation (Additional file 1). Based on histopathology and immunohistochemistry (CD20, CD3), tumors were classified according to the modified WHO criteria [9].

Also, dogs were only included, if information regarding treatment and outcome were complete.

As this evaluation did not influence any therapeutic decision, approval by an Ethics Committee was not required. Owners gave their written informed consent to the use of clinical data.

### Treatment protocol

#### Vaccinated dogs

The detailed method of vaccine preparation and the protocol used for dogs treated with chemo-immunotherapy has been described elsewhere, [[7] Additional file 1].

Briefly, dogs received l-asparaginase, vincristine, cyclophosphamide, doxorubicin, lomustine, prednisone, and a total of 8 intradermal injections of 0.5 ml vaccine.

For immunological monitoring, the DTH skin test was performed in all vaccinated dogs at the end of treatment (Additional file 1).

#### Unvaccinated dogs

Unvaccinated dogs received two CHOP-based protocol, including l-asparaginase, vincristine, cyclophosphamide, doxorubicin, prednisone, with or without lomustine(Additional file 1); the intended summation dose intensity of these two protocols was 16.8 and 16.9, respectively. Thus, they were considered identical and evaluated as a whole.

#### Response assessment and minimal residual disease monitoring

Response was evaluated at each treatment session according to previously published criteria [10].

Two weeks after having completed the protocol, all dogs underwent restaging by repeating the initially altered examination. For minimal residual disease monitoring, FC on PB, BM and a LN aspirate was carried out [11, 12]. Dogs were then rechecked through monthly physical examinations during the first year, and every other month thereafter.

Relapse was defined as clinical reappearance and cytological evidence of lymphoma in any anatomical site in dogs having experienced CR, whereas relapse for animals with PR was defined as progression.

Dogs that relapsed during or after treatment were offered rescue chemotherapy.

### Endpoints

The primary study objectives were the effectiveness of chemo-immunotherapy in dogs with B-cell lymphoma, measured as TTP, LSS, and 1-, 2-, and 3-year survival rates. This was evaluated in the whole population and by stratifying dogs according to potential prognostic variables, in order to select the best candidates for chemo-immunotherapy.

The secondary objective was safety (measured by recording any AE and/or hospitalization that occurred during or immediately after treatment). All AEs were registered at the time of occurrence, and graded according to VCOG [13].

### Statistical analysis

Descriptive statistics were used in the analysis of dogs and tumor characteristics. The distribution of demographic features and possible outcome variables between treatment groups were assessed with Fisher’s exact test/χ2 test (categorical variables).

Variables considered were: sex, age, weight, PCV, platelet count, serum LDH activity, serum Ionized calcium concentration, substage, PB infiltration, BM infiltration, extranodal site involvement, and pre-treatment with steroids.

TTP was calculated as the interval between initiation of treatment and PD or relapse; dogs with no PD or relapse at data-analysis closure or death were censored. LSS was measured as the interval between initiation of treatment and death or euthanasia for lymphoma or chemo-related causes. Dogs deceased for lymphoma-unrelated causes or alive at data-analysis closure were censored. Survival plots were generated according to the Kaplan-Meier product-limit method. Curves were compared with the log-rank test. The influence of potential prognostic variables on tumor progression and tumor-related death was investigated with univariable and multivariable Cox’s regression analysis.

The survival advantage of vaccine administration was further tested upon stratification of cases according to the other considered variables. The variables with no survival advantage for one of the strata were selected for a scoring system to quantify the utility of vaccine administration. Dogs with the highest score were the best candidates for immunotherapy.

Data were analyzed by use of commercial software programs (SPSS Statistics v19, IBM, Armonk, NY, USA, Prism v.5.0, GraphPad, San Diego, California). *P*-values < 0.05 were considered significant.

## Results

The total population consisted of 300 dogs: 246 (82%) underwent lymphadenectomy, whereas in the remaining 54 (18%) a cytological and FC diagnosis only was obtained.

Within the histology group, DLBCL was the most common histotype (*n* = 148; 60.2%), followed by nodal MZL late stage (*n* = 49; 19.9%), FL (*n* = 25; 10.2%), Burkitt lymphoma (*n* = 10; 4.1%), SLL (*n* = 8; 3.3%), and lymphoblastic lymphoma (*n* = 6; 2.4%).

Within the cytology group, there were 50 (92.6%%) centroblastic/immunoblastic lymphomas, 2 (3.7%) lymphoblastic lymphomas, and 2 (3.7%) medium macronucleolated cell lymphomas.

Within the whole population, 148 (49.3%) dogs received chemotherapy alone and 152 (50.7%) chemo-immunotherapy.

Median TTP was 147 days (95% CI, 111–183) for dogs receiving chemotherapy and 244 days (95% CI, 218–270) for those receiving chemo-immunotherapy (*P* <  0.001). Median LSS was 220 days (95% CI, 157–243) for dogs receiving chemotherapy and 401 days (95% CI, 339–463) for those receiving chemo-immunotherapy (*P* < 0.001).

The three main histotypes are detailed in the following paragraphs.

### Dogs with diffuse large B-cell lymphoma

Table 1 provides a summary of the characteristics of 148 dogs with DLBCL using known or potential covariates for outcome.

**Table 1:** Baseline characteristics of 222 dogs with B-cell lymphoma treated with chemotherapy alone or with chemo-immunotherapy and stratified according to histotype

Variable	DLBCL (*n* = 148)			MZL (*n* = 49)			Follicular lymphoma (*n* = 25)		
	CH (*n* = 47)	CH-IT (*n* = 101)	*P*	CH (*n* = 24)	CH-IT (*n* = 25)	*P*	CH (*n* = 6)	CH-IT (*n* = 19)	*P*
Sex			0.522			0.484			0.175
Male	25	48		12	15		4	6	
Female	22	53		12	10		2	13	
Median age (range) - *years*	9 (3–12)	7 (3–15)	0.677	8 (3–15)	7 (3–13)	0.110	10 (6–11)	9 (7–11)	0.774
Median weight (range) - *kg*	31.9 (4.1–60.0)	28.8 (17–38)	0.787	20.7 (3.0–42.3)	26.0 (3.9–44.4)	0.556	29 (20–40)	20 (12–32)	0.171
PCV			0.063			0.189			0.430
normal	46	89		20	24		5	18	
decreased	1	12		4	1		1	1	
Platelet count			0.127			0.138			0.999
normal	34	84		18	23		5	17	
decreased	13	17		6	2		1	2	
LDH			0.689			0.567			0.999
normal	23	53		15	13		4	11	
increased	24	48		9	12		2	8	
Stage			0.310			0.999			0.999
III-IV	15	41		4	4		2	8	
V	32	60		20	21		4	11	
Peripheral blood infiltration			0.129			0.496			0.160
no	17	50		6	4		5	8	
yes	30	51		18	21		1	11	
Bone marrow infiltration			0.647			0.791			0.999
no	20	47		5	6		3	9	
yes	27	54		19	19		3	10	
Extranodal involvement			0.004*			0.680			0.562
no	33	90		17	19		4	16	
yes	14	11		7	6		2	3	
Substage			0.198			0.484			0.999
a	27	69		14	17		5	14	
b	20	32		10	8		1	5	
Steroids before referral			0.284			0.038*			0.540
no	37	71		15	22		6	15	
yes	10	30		9	3		0	4	
Rescue protocols			0.275			0.696			0.400
untreated	1	8		1	1		1	1	
one RP administered	10	21		3	8		1	5	
more RP administered	5	23		4	5		0	2	
Toxicity			0.167			0.667			0.211
none/grades 1–2	39	85		21	23		3	15	
grades 3–4	5	15		3	2		2	3	
grade 5	3	1		0	0		1	1	

Abbreviations: *DLBCL* Diffuse large B-cell lymphoma, *MZL* marginal zone lymphoma, *PCV* Packed cell volume, *LDH* Lactate dehydrogenase, *CH* Chemotherapy, *CH-IT* Chemo-immunotherapy, *RP* Rescue protocol

*Significant

Forty (27%) dogs had been treated with steroids before referral. Forty-seven (31.8%) were treated with chemotherapy and 101 (68.2%) received chemo-immunotherapy. There was a good balance between treatment arms regarding demographic information and possible outcome variables, including the number of rescue protocols, with the exception of a lower percentage of dogs with extranodal involvement in the chemo-immunotherapy group (Table 1).

Median TTP was 98 days (95% CI, 9–187) for dogs receiving chemotherapy and 250 days (95% CI, 210–290) for those receiving chemo-immunotherapy (*P* = 0.001). Median LSS was 165 days (95% CI, 107–223) for dogs receiving chemotherapy and 413 days (95% CI, 316–510) for those receiving chemo-immunotherapy (*P* = 0.001).

There was no significant difference in TTP and LSS between dogs with positive and negative DTH test.

The 1-year, 2-year and 3-year survival rates were, respectively, 20, 13 and 8% for the chemotherapy group, and 51, 19 and 10% for the chemo-immunotherapy group.

On multivariable analysis, lack of immunotherapy administration was significantly associated with an increased risk of tumor progression (HR = 2.3, 95% CI = 1.4–3.6, *P* = 0.001) and tumor-related death (HR = 2.6, 95% CI = 1.6–4.2, *P* < 0.001). The benefit of chemo-immunotherapy was particularly relevant in dogs with high serum LDH levels, stage V disease, substage a, and not previously treated with steroids.

**Fig. 1:**
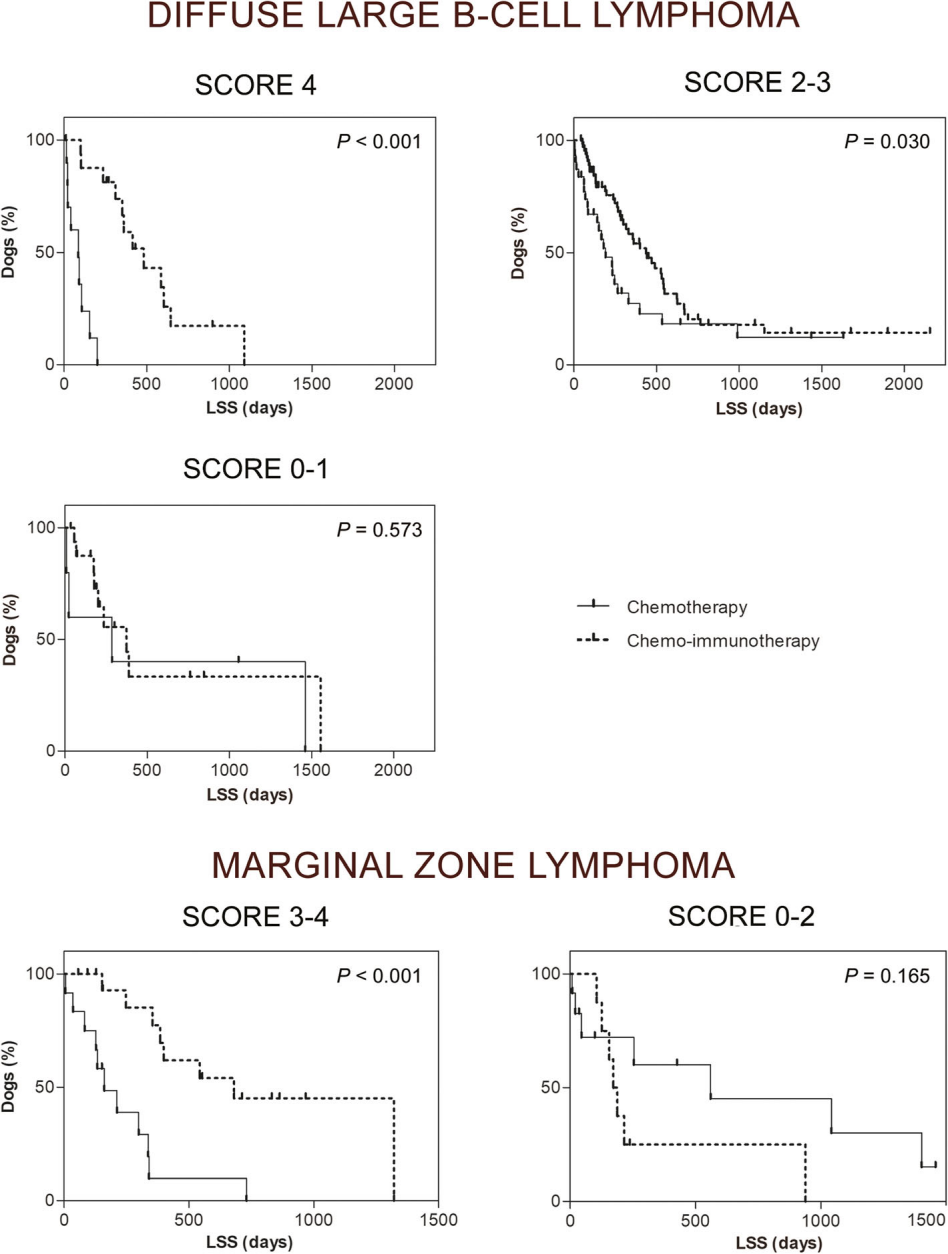
Survival curves of dogs with DLBCL and MZL grouped according to the proposed score

The patients with all of the above characteristics obtained the greatest survival advantage from the administration of immunotherapy (median LSS, 480 vs 85 days; *P* < 0.001). Among dogs falling in two or three of the above categories, those treated with chemo-immunotherapy still had a significantly better outcome, but the survival advantage was reduced (median LSS, 435 vs 190 days; log-rank, *P* = 0.030). Finally, dogs falling in one or none of the above categories did not receive any significant benefit from immunotherapy (median LSS, 374 vs 286 days, log-rank, *P* = 0.573; Table 2; Fig. 1).

**Table 2:** Score to evaluate the benefits of the treatment with chemo-immunotherapy in dogs with DLBCL and MZL

*Serum LDH levels*		Normal (0)			Increased (+ 1)				
*Stage*		III/IV (0)			V (+ 1)				
*Substage*		b (0)			a (+ 1)				
*Steroids before referral*		Yes (0)			No (+ 1)				
Dogs with diffuse large B-cell lymphoma									
Score	Median LSS			1-year SR		2-year SR		3-year SR	
	CH	CH-IM	*P*	CH	CH-IM	CH	CH-IM	CH	CH-IM
4 (*n* = 27)	85	480	< 0.001*	0%	57%	0%	15%	0%	0%
2–3 (*n* = 99)	190	435	0.030*	23%	50%	12%	18%	8%	11%
0–1 (n = 22)	286	374	0.573	40%	45%	40%	27%	25%	11%
All cases (n = 148)	165	413	0.001*	20%	51%	13%	19%	8%	10%
Dogs with marginal zone lymphoma									
Score	Median LSS			1-year SR		2-year SR		3-year SR	
	CH	CH-IM	*P*	CH	CH-IM	CH	CH-IM	CH	CH-IM
3–4 (*n* = 29)	160	680	< 0.001*	9%	77%	0%	36%	0%	12%
0–2 (*n* = 20)	560	172	0.165	56%	14%	38%	14%	25%	0%
All cases (n = 49)	254	399	0.245	30%	55%	16%	28%	10%	10%

Abbreviations: LDH, lactate dehydrogenase; LSS, lymphoma specific survival; SR, survival rate; CH, chemotherapy; CH-IM, chemo-immunotherapy

* Significant

### Dogs with nodal marginal zone lymphoma

Table 1 provides a summary of the characteristics of 49 dogs with MZL using known or potential covariates for outcome.

Twelve (24.5%) dogs had been treated with steroids before referral.

Twenty-four (48.9%) dogs received chemotherapy alone and 25 (51.1%) chemo-immunotherapy. Dogs were evenly balanced between groups according to possible outcome variables, including the number of rescue protocols, with the exception of a lower percentage of dogs receiving steroids before being treated with chemo-immunotherapy (Table 1).

Median TTP was 147 days (95% CI, 47–247) for dogs receiving chemotherapy and 227 days (95% CI, 165–289) for those receiving chemo-immunotherapy (*P* = 0.082). Median LSS was 254 days (95% CI, 69–442) for dogs receiving chemotherapy and 399 days (95% CI, 133–665) for those receiving chemo-immunotherapy (*P* = 0.245).

There was no significant difference in TTP and LSS between dogs with positive and negative DTH test.

The 1-year, 2-year and 3-year survival rates were, respectively, 30, 16 and 10% for the chemotherapy group and 55, 28 and 10% for the chemo-immunotherapy group.

When including in the survival analysis only the dogs falling in at least three of the 4 categories identified for DLBCL, the survival advantage of chemo-immunotherapy was statistically significant (median LSS, 680 vs 160 days, log-rank, *P* < 0.001; Table 2; Fig. 1).

### Dogs with follicular lymphoma

Among the 25 dogs with FL, 4 (16%) had been treated with steroids before being referred.

Six (24%) dogs were treated with chemotherapy and 19 (76%) received chemo-immunotherapy. There was a good balance between treatment arms regarding demographic information and possible outcome variables, including the number of rescue protocols (Table 1).

Median TTP was 168 days (95% CI, 1–386) for dogs receiving chemotherapy and 273 days for those receiving chemo-immunotherapy (*P* = 0.076). Median LSS was 200 days (95% CI, 1–462) for dogs receiving chemotherapy and 436 days (95% CI, 201–671) for those receiving chemo-immunotherapy (*P* = 0.011).

The 1-year, 2-year and 3-year survival rates were 0% for the chemotherapy group, and 36, 27 and 18% for the chemo-immunotherapy group.

On multivariable survival analysis, lack of immunotherapy was the only variable significantly associated with increased risk of tumor-related death (HR = 5.2; 95% CI = 1.1–25.2; *P* = 0.039).

### Toxicity

All dogs included in the study were evaluable for toxicity.

Immunotherapy was well tolerated, with no reported local or systemic AEs.

Chemotherapy was similarly well tolerated. There was no significant difference among groups regarding grade and frequency of AEs (Table 1).

## Discussion

Immunotherapy is increasingly acknowledged as an effective treatment for several canine cancers including, malignant melanoma, B-cell lymphoma, osteosarcoma, and brain tumors [7, 8, 14–22]. Studies in the field have unfortunately shown that not all dogs will have a substantial benefit, and it is incumbent upon clinicians to decide whether a dog is or is not a candidate for immunotherapy.

Herein, we present the results of a study aimed at comparing the outcomes of dogs with B-cell lymphoma receiving chemotherapy or chemo-immunotherapy. The primary goal of this study was to identify the candidate dogs that would benefit the most from chemo-immunotherapy in the standard daily clinical practice outside a trial setting. So far only two small clinical trials on chemo-immunotherapy have been reported [7, 8]. The current study ranks among the largest per number of dogs treated with the same product for a specific disease and confirms the efficacy and the good safety profile of this treatment.

It was herein confirmed that DLBCL is the most common histotype in dogs, followed by MZL and FL. By analyzing the whole population it was also confirmed that stage III disease is quite infrequent, whereas stage V was most commonly diagnosed (70.7%). It is likely that an accurate staging leads to stage migration, having prognostic and therapeutic implications, as documented by the current data.

Overall, the present study demonstrates a significant clinical benefit of immunotherapy. In particular, subgroup analysis of LSS, comparing treatment arms using a multivariable method, indicated the following.

It was found that immunotherapy conferred a survival benefit in the majority of DLBCL cases. However, dogs that benefited the most were those with stage V disease, no systemic symptoms, a high serum LDH, and not previously treated with steroids. Interestingly, if these characteristics were concurrent, vaccinated dogs had the highest survival advantage (480 vs 85 days, respectively).

If two or three of the abovementioned characteristics were present, the survival benefit for vaccinated dogs was still significant, although to a lesser extent (435 vs 190 days, respectively). If only one or none of the abovementioned characteristics were present, there was no demonstrable benefit of chemo-immunotherapy over chemotherapy, although these cases represented a minority (*n* = 22).

It could be hypothesized that the stimulation of the immune system may contribute to counteract the negative impact of well-known prognostic factors that commonly lead to a poor outcome if chemotherapy only is administered. While chemotherapy effects only last as long as the drugs remain in the body, immunotherapy can provide long-term protection against cancer due to the immune memory, overcoming the “honeymoon effect” provided in the short term by cytotoxic drugs, more over if negative prognostic factors are present. Chemotherapy fails when drug-resistant clones emerge, preventing tumor cell eradication [23]. It may be possible that dogs with negative prognostic factors, such as advanced disease stage and high LDH levels, are more susceptible of developing chemo-resistance [2, 24, 25]. In these dogs, chemo-resistant clones may be eliminated by cell-mediated immunotherapy because they can evade neither immune surveillance nor immune response, thereby providing a survival benefit.

Finally, the lower immunity in symptomatic dogs is a plausible explanation to the suboptimal response to immunotherapy, and specific immune responses may be abrogated by pre-treatment steroids.

These findings were also confirmed for the MZL cases: vaccinated dogs survived significantly longer than unvaccinated dogs (680 vs 160 days, respectively; *P* < 0.001) if three or four of the previously identified characteristics were present. If 2 to none of those were present, there was no significant benefit in administering chemo-immunotherapy over chemotherapy.

In dogs with FL, a survival benefit of vaccine-administration was demonstrated as well, however the population was too small to evaluate the potential inference of other prognostic factors.

Finally, it must be reminded that, although chemo-immunotherapy seems to provide better results than chemotherapy for the majority canine B-cell lymphomas, the 3-year survival rate remains largely unsatisfactory, ranging from 0 to 12%; whereas the largest survival benefit can be experienced in the short/medium-term period. It is currently unknown whether re-vaccination or preparation of a new vaccine starting from nodal relapses would improve long-term survival.

Our study has a number of limitations, such as the small number of dogs included in some of the categories and its retrospective nature. However, we have included consecutive dogs diagnosed following a uniform approach and treated with standardized protocols.

Third, the evaluation of the immune response by the DTH skin test, albeit clinically relevant, is operator-dependent. Standardized and quantitative analyses are warranted to reduce this constraint.

Last, LSS may have been influenced by tumor’s unrelated factors, including owners’ motivation and financial concern. However, almost all dogs in both treatment arms received at least one rescue protocol, thereby reducing the risk of bias.

## Conclusions

Our study shows a response and survival benefit of the addition of active immunotherapy to chemotherapy in dogs with B-cell lymphoma, possibly due to a different and perhaps synergistic mechanism of action. In the future, the development of new effective immunotherapeutic strategies should take into account differences in immune microenvironment between different lymphoma molecular subtypes to find the best treatment for each patient. A large-scale, double-blinded, randomized, multi-institutional trial is essential for ascertaining the efficacy of the presently described active immunotherapy procedure and its clinical application.

## Additional file

Additional file 1: Supplementary data. (DOCX 105 kb)

## Data Availability

All data generated or analysed during this study are included in this published article [and its Additional file].

## Abbreviations

AE: Adverse event
BM: Bone marrow
CI: Confidence interval
CR: Complete remission
DLBCL: Diffuse large B-cell lymphoma
DTH: Delayed-time hypersensitivity
FC: Flow cytometry
FL: Follicular lymphoma
HSPs: Heat Shock Proteins
LDH: Lactate dehydrogenase
LN: Lymph node
LSS: Lymphoma-specific survival
MZL: Marginal zone lymphoma
ORR: Overall response rate
PB: Peripheral blood
PCV: Packed cell volume
PR: Partial remission
SLL: Small lymphocytic lymphoma
TTP: Time to progression
