# Supplementary material for: Opportunities and challenges of active immunotherapy in dogs with B-cell lymphoma: a 5-year experience in two veterinary oncology centers
Source: J Immunother Cancer. 2019 Jun 7;7:146. doi: 10.1186/s40425-019-0624-y (PMC6554898; doi:10.1186/s40425-019-0624-y)
Supplement: Supplementary file 1 — Supplementary data. (DOCX 105 kb) [file 40425_2019_624_MOESM1_ESM.docx]

**Supplementary data**

**Staging work-up**

To be eligible for inclusion in the analysis, dogs were required to undergo a complete initial staging work-up, consisting of history and physical examination, complete blood cell count with differential, serum biochemistry profile including LDH, thoracic radiographs, abdominal ultrasound, cytological evaluation of liver and spleen regardless of their sonographic appearance, FC on LN, PB and BM, and, whenever possible, being compatible with the dogs’ condition and owners’ compliance, surgical removal of a peripheral LN to obtain a histopathological diagnosis and material for the vaccine generation.

**Autologous vaccine preparation**

Intracellular proteins were extracted from the tumor sample by homogenisation and centrifugation (5000g for 10 mn) to remove cell debris. The supernatant was then diluted in a supersaturated ammonium sulfate solution (50/50 v/v). The proteins were precipitated for 1h at 4°C, then the solution was centrifuged at 5000g (30 mn). The clot was re-suspended in a phosphate buffer (pH 6.8, 20mM). Then, this solution was passed through a column containing hydroxyapatite powder (350 mg). The powder was washed using a phosphate buffer solution (pH 6.8, 20 mM). Finally, the powder was suspended in a 2% carboxymethyl cellulose solution and divided in 8 syringes of 1 ml each. Each dose of vaccine contained 0.5 ml NaCl, 0.02M (pH 6.8, 20 mM) carboxymethylcellulose 2%, 41 mg hydroxyapatite powder, <1500 µg HSP.

Quality controls included SDS page and dot blot. For SDS page, the proteins were recovered from the powder by washing 10 mgrs of vaccine powders in 0.5 ml of a 0.5 M NaCl solution. The SDS was then made using tris-glycine gels (in vitrogen) according to manufacturer’s instructions. For dot blot, 10 µl of the vaccine solution were deposited on a nitrocellulose membrane and allowed to dry at open air. After drying, the membrane was washed with a blocking solution (1% BSA in PBS) and then with a Tris buffer solution (pH 7.4) containing 0.5% tween detergent 20. The membrane was then incubated with the primary antibody for one hour at room temperature (anti-gp96, anti-HSP70, stressgen-US) before being washed in the same buffer. The membrane was incubated for 30 minutes with the secondary antibody labeled with an alkaline phosphatase and then washed with the buffer before adding an alkaline phosphatase developer.

**Immunological monitoring**

For immunological monitoring, the DTH skin test was performed in all vaccinated dogs at the end of treatment and the results were read by a trained veterinarian. Briefly, the tumor extract to be injected in the dermis for DTH skin test was prepared as follows: 100 mL of the autologous tumor homogenate was diluted in 0.5 mL of a CMC solution (2% in 20 mmol/L NaCl); 0.1 mL of the solution obtained was injected intradermally into a different site from the vaccination site. The presence of erythema or induration was recorded 24 and 48 h after the injection. The test was considered positive if the area of induration and/or erythema was more than 10 mm in diameter.

**CHOP-based protocols administered to unvaccinated dogs**

*Protocol 1 (dose intensity 16.8):*

Week 1: L-Asparaginase @ 400 UI/kg SC

Week 2: Vincristine @ 0,75 mg/m2 IV

Week 3: Cyclophosphamide @ 75 mg/m2 PO for 4 consecutive days

Week 4: Doxorubicin @ 30 mg/m2 IV

Week 5: Vincristine

Week 6: Cyclophosphamide @ 75 mg/m2 PO for 4 consecutive days

Week 7: Doxorubicin @ 30 mg/m2 IV

Week 8: rest

Week 9: Lomustine @ 80 mg/m2 PO

Week 10: rest

Week 11: Vincristine @ 0,75 mg/m2 IV

Week 12: Cyclophosphamide @ 75 mg/m2 PO for 4 consecutive days

Week 13: Doxorubicin @ 30 mg/m2 IV

Week 14: rest

Week 15: Lomustine @ 80 mg/m2 PO

Week 16: rest

Week 17: Vincristine @ 0,75 mg/m2 IV

Week 18: Cyclophosphamide @ 75 mg/m2 PO for 4 consecutive days

Week 19: Doxorubicin @ 30 mg/m2 IV

Prednisolone administered at 1 mg/kg daily from week 1 to week 4; then 0.5 mg/kg daily until the end of treatment

*Protocol 2 (dose intensity 16.9) (Simon et al, JVIM 2006)*

Week 1: L-Asparaginase @ 400 UI/kg SC and Vincristine @ 0,7 mg/m2 IV

Week 2: Cyclophosphamide @ 200 mg/m2 IV

Week 3: Doxorubicin @ 30 mg/m2 IV

Week 4: Vincristine @ 0,7 mg/m2 IV

Week 5: Cyclophosphamide @ 200 mg/m2 IV

Week 6: Doxorubicin @ 30 mg/m2 IV

Week 7: Vincristine @ 0,7 mg/m2 IV

Week 8: Cyclophosphamide @ 200 mg/m2 IV

Week 9: Doxorubicin @ 30 mg/m2 IV

Week 10: Vincristine @ 0,7 mg/m2 IV

Week 11: Cyclophosphamide @ 200 mg/m2 IV

Week 12: Doxorubicin @ 30 mg/m2 IV

Prednisolone administered at 50 mg/m2 PO on days 1-3 of every treatment week
